# A transgenic mouse line for assaying tissue-specific changes in endoplasmic reticulum proteostasis

**DOI:** 10.1007/s11248-023-00349-7

**Published:** 2023-05-03

**Authors:** Reinis Svarcbahs, Sarah M. Blossom, Helena S. Baffoe-Bonnie, Kathleen A. Trychta, Lacey K. Greer, James Pickel, Mark J. Henderson, Brandon K. Harvey

**Affiliations:** 1 Cellular Stress and Inflammation Section, Intramural Research Program, National Institute On Drug Abuse, National Institutes of Health, Baltimore, MD 21224 USA; 2 National Center for Advancing Translational Sciences, National Institutes of Health, Rockville, MD 20850 USA; 3 Transgenic Technology Core, Intramural Research Program, National Institute of Mental Health, Bethesda, MD 20892 USA

**Keywords:** Exodosis, ER calcium sensor, ER stress, *Gaussia* luciferase, SERCaMP, Secreted ER-resident proteins

## Abstract

Maintenance of calcium homeostasis is important for proper endoplasmic reticulum (ER) function. When cellular stress conditions deplete the high concentration of calcium in the ER, ER-resident proteins are secreted into the extracellular space in a process called exodosis. Monitoring exodosis provides insight into changes in ER homeostasis and proteostasis resulting from cellular stress associated with ER calcium dysregulation. To monitor cell-type specific exodosis in the intact animal, we created a transgenic mouse line with a *Gaussia* luciferase (GLuc)—based, secreted ER calcium-modulated protein, SERCaMP, preceded by a LoxP-STOP-LoxP (LSL) sequence. The Cre-dependent LSL-SERCaMP mice were crossed with albumin (Alb)-Cre and dopamine transporter (DAT)-Cre mouse lines. GLuc-SERCaMP expression was characterized in mouse organs and extracellular fluids, and the secretion of GLuc-SERCaMP in response to cellular stress was monitored following pharmacological depletion of ER calcium. In LSL-SERCaMP × Alb-Cre mice, robust GLuc activity was observed only in the liver and blood, whereas in LSL-SERCaMP × DAT-Cre mice, GLuc activity was seen in midbrain dopaminergic neurons and tissue samples innervated by dopaminergic projections. After calcium depletion, we saw increased GLuc signal in the plasma and cerebrospinal fluid collected from the Alb-Cre and DAT-Cre crosses, respectively. This mouse model can be used to investigate the secretion of ER-resident proteins from specific cell and tissue types during disease pathogenesis and may aid in the identification of therapeutics and biomarkers of disease.

**Supplementary Information:**

The online version contains supplementary material available at 10.1007/s11248-023-00349-7.

## Introduction

Endoplasmic reticulum (ER) proteostasis is important for protein synthesis, folding, transport, and secretion with almost one-third of the proteome synthesized at the ER (Jan et al. 2014; Martínez et al. 2017). Additionally, ER proteostasis is tightly linked to ER calcium concentrations. Pharmacological interventions, physiological cues, and pathological conditions that deplete ER calcium disrupts ER proteostasis and activate the unfolded protein response (Sans et al. 2002, Preissler et al. 2020). Under normal conditions, the ER maintains calcium at a concentration of 1 to 3 mM, 1000 to 10,000 times greater than the calcium concentration of the cytosol (Meldolesi and Pozzan 1998). The steep calcium gradient between the ER and cytosol is regulated by a series of pumps and channels (Michalak et al. 2002; Brandman et al. 2007). The Sarco/Endoplasmic Reticulum Calcium ATPase (SERCA) pump actively transports cytosolic calcium against the gradient into the ER while the inositol-1,4,5-trisphosphate receptor (IP3R) and the ryanodine receptor (RyR) release calcium from the ER stores (Krebs et al. 2015, Primeau et al. 2018). Disrupted ER calcium homeostasis and the subsequent protein misfolding has been implicated in a variety of pathologies ranging from diabetes mellitus (Laybutt et al. 2007) to Alzheimer’s and Parkinson’s disease (Chan et al. 2009; Demuro et al. 2010).

Since ER calcium homeostasis is crucial to maintaining a healthy cellular state, techniques have been created to study calcium homeostasis. Intracellular calcium imaging technology began with fast-acting synthetic dyes, such as Fura-2, that react in the presence of calcium (Grynkiewicz et al. 1985). Subsequently, genetically encoded calcium indicators (GECIs), including the fluorescent protein GCaMP, were engineered and improved upon to measure changes in calcium via fluorescence imaging (Lin and Schnitzer 2016, Inoue 2021). However, these approaches are limited to detecting acute changes in calcium homeostasis and are unable to provide information about changes in ER proteostasis. To circumvent this limitation in vivo, we used a *Gaussia* luciferase (GLuc)—based, secreted ER calcium-modulated protein, SERCaMP, which reflects ER-resident protein behavior and acts as an indirect indicator of ER calcium homeostasis (Henderson et al. 2014). Previous work from our lab demonstrates a phenomenon whereby ER-resident proteins are secreted following a depletion in ER calcium, coined “exodosis” (Trychta et al. 2018). By measuring extracellular GLuc activity, such as GLuc in body fluids, the reporter levels can reflect changes in ER proteostasis resulting from altered ER calcium depletion. For example, previous work demonstrated that a high fat diet increased GLuc activity in the plasma from rats that received an intra-liver injection of AAV-SERCaMP. Here, we describe the creation and characterization of a Cre-dependent SERCaMP transgenic mouse to facilitate cell- and tissue- specific monitoring of exodosis. We targeted the mouse ROSA26 safe harbor locus with a LoxP-STOP-LoxP (LSL) cassette where the transcriptional “STOP” sequence restricts reporter expression unless Cre is present (Agarwal et al. 2017). By measuring GLuc activity in extracellular fluids (e.g., cerebrospinal fluid (CSF) or blood plasma) we are able to monitor exodosis over time.

To test our new Rosa26-LoxP-STOP-LoxP-GLuc-SERCaMP “LSL-SERCaMP” reporter mouse for the ability to report on cell/tissue-specific exodosis, we crossed it with two different Cre-driver lines. We used an albumin (Alb)-Cre mouse to limit expression predominantly to liver hepatocytes and a dopamine transporter (DAT)-Cre mouse to express SERCaMP reporter in dopaminergic neurons in the brain. We demonstrate that (1) SERCaMP expression is localized to the intended tissues in these transgenic mice and (2) the reporter responds to perturbations in ER calcium, which can be assayed by collecting biological fluids. The LSL-SERCaMP reporter mouse can be used to identify temporal changes in ER proteostasis in vivo.

## Materials and methods

### Vector construction

Construction of pAAV1 CMV-IE Nuc-IRFP-2A-iCre (Addgene plasmid #112,683) was previously described (Bäck et al. 2019). AAV was packaged, purified and titered as previously described (Howard and Harvey 2017). For generation of SERCaMP mouse line, the coding sequence of GLuc-ASARTDL (SERCaMP) was amplified from pdsAAV-CMV-GLUC-ASARTDL (SERCaMP) plasmid (Henderson et al. 2014) and inserted used to replace mGCaMP3 in the Rosa26-lsl-mGCaMP3 plasmid, a kind gift from Dr. Dwight E. Bergles (Agarwal et al. 2017). Briefly, backbone was digested with MluI (#R0198S; NEB) and EcoRV (R0195S; NEB), gel purified and recombined with inserts using an In-Fusion HD cloning kit (639,645, Clontech). Resulting targeting construct was sequence verified.

### Generation of mouse line

A fragment containing ROSA26 homology arms, CAG promoter, Lox-STOP-LoxP, MANFsp-GLUC-ASARTDL and FRT flanked Neomycin gene was excised from the construct at KflI and ClaI sites. DNA was prepared using endotoxin free reagents. It was electroporated into C57 Bl6/N ES lines. Clones were plated on antibiotic resistant feeder cells and selected with 200 µg/mL G418. One hundred fifty surviving clones were picked, expanded, and screened for the proper insertion of the transgene using PCR from the 5ʹ and 3ʹ ends of the insert into the flanking target site. Five clones with the proper insertion were expanded and injected into blastocyst stage embryos which were surgically transferred into the oviducts of outbred foster dams. Founder pups were selected for those with the highest percent of ES cell encoded coat color or GFP fluorescence. Germ-line transmission of these markers and the proper transgene insertion were demonstrated for two clones which were used for following experiments.

### Animals

Animal studies were approved by the NIDA and NIMH Animal Care and Use Committees (ACUC) and comply with NIH guidelines for animal research. Mice were maintained in C57BL/6 J background. Mice were group housed under standard laboratory conditions (12 h light/dark cycle; room temperature, 23 ± 2 °C; relative humidity, 50 ± 15%) in individually ventilated cages. Both sexes were used in all experiments.

After identification of LSL-SERCaMP founder mice, LSL-SERCaMP mice were crossed with ACTB-Flp (B6.Cg-Tg(ACTFLPe)9205Dym/J; Stock No: 005703) mice to remove Neomycin gene from the mROSA26 locus. LSL-SERCaMP mice were backcrossed for 15 generations with C57BL/6 J mice after removing Neomycin cassette. LSL-SERCaMP mice were crossed with either DAT-Cre (B6.SJL-Slc6a3tm1.1(cre)Bkmn/J; RRID:IMSR_JAX:006,660) (Bäckman et al. 2006) or Alb-Cre (B6.Cg-Speer6-ps1Tg(Alb-cre)21Mgn/J; RRID:IMSR_JAX:003,574) reporter line and double positive genotypes, LSL-SERCaMP, Alb-Cre, DAT-Cre and wildtype littermates were used in experiments. The GLuc-SERCaMP mouse is registered with Mouse Genome Institute as strain C57BL/6J-Tg(CAG-gluc*)1Harv/Harv MGI:7466789. DAT-Cre and Alb-Cre mice were crossed with tdTomato Ai14 (B6.Cg-Gt(ROSA)26Sortm14(CAG-tdTomato)Hze/J; RRID:IMSR_JAX:007,914) line.

### Surgery

Animal studies were approved by the NIDA Animal Care and Use Committee and comply with NIH guidelines for animal research. AAV1 CMV-IE Nuc-IRFP-2A-iCre (2 µL at 0.5 µL, viral titer: 2.25E + 11 vg/mL) was injected unilaterally in SERCaMP mouse striatum (coordinates, A/P: 0.7, M/L: 1.8, D/V: − 3.0). Two weeks post-injection mice were deeply anesthetized and perfused with either 0.9% saline for tissue homogenization or with 0.9% saline followed by 4% paraformaldehyde for immunohistochemistry analyses.

For CSF collection, 26G injection cannula (#C315GAS, P1 Technologies, Roanoke, Virginia) was implanted in mouse ventricle (coordinates: A/P: − 0.2; M/L: 0.9; D/V: − 2.0). After the cannulation of SERCaMP × DAT-Cre mouse lateral ventricle, mice were housed individually and allowed to recover for two weeks. Up to 10 µL of CSF was collected weekly by lowering blunt 33G needle attached to 10 µL microinjection syringe into the cannula and slowly withdrawing CSF at 1 µL/min with microinjector (WPI). For terminal CSF collection, adapted protocol was used (Liu and Duff 2008). Briefly, after anesthetizing mouse was placed in stereotaxic device. After skin incision, cisterna magna was uncovered. 26G needle was attached to tubing connected to 100 µL syringe. Needle was placed into the probe holder with bevel upward. After needle was positioned over the cisterna magna, cisterna magna was punctured with the needle and CSF was withdrawn at 1 µL/min using microinjector (WPI).

### Thapsigargin (TG) injections

TG (#T9033, Millipore-Sigma) was administered by i.p. injections at 0.7 mg/kg for male and 0.5 mg/kg for female mice. Concentrations were selected based on (Abdullahi et al. 2017) and our own observation that female mice are less tolerant to TG injections. TG powder was dissolved to a final concentration of 1 mg/mL in absolute ethanol. Solutions were prepared by diluting stock solution in sterile saline prior to injections.

### Immunohistochemistry

The animals were perfused with PBS with heparin followed by 4% paraformaldehyde. Subsequently, livers and brains were removed and transferred to 4% paraformaldehyde followed by 18% and 30% sucrose gradient until equilibrated, as evidenced by sinking to the bottom of tube. Tissue was flash frozen on dry ice. Frozen brains and livers were sectioned with a cryostat (Leica CM1950) at 30 µm. The sections were stained with rabbit anti-GLuc (1:2000; NEB, #E8023S), chicken anti-albumin Sigma SAB3500217 (1:200; Sigma-Aldrich, #SAB3500217; RRID:AB_10640041), mouse anti-tyrosine hydroxylase (1:2000; Millipore, #MAB318; RRID:AB_2201528), chicken anti-calreticulin (1:200; Invitrogen, PA1-902A; RRID:AB_2069607), and rabbit anti-MANF (1:1000; custom polyclonal, Yenzyme, #YZ2155). Nuclei were stained with DAPI (4ʹ,6-Diamidino-2-Phenylindole, Dilactate, 1:3000; Invitrogen, #D3571) then mounted on glass slides with Mowiol. Sections were imaged and captured with a Nikon C1 confocal microscope and processed in Fiji-ImageJ.

### Blood sampling and plasma separation

For terminal bleed, mice were deeply anesthetized with isoflurane saturated in air. Laparotomies were performed to expose abdominal aorta. Using a 25G needle and 1 mL syringe, blood was slowly withdrawn from the abdominal aorta. The blood samples were then transferred to pre-weighed 1.5 mL centrifuge tubes that contained 50 µL of heparin (1000 U/mL; Sagent Pharmaceuticals, #NDC 25021-400-30). The blood samples were kept on ice until they were processed but no longer than 90 min. The blood to heparin ratio was normalized to be 2:1. The heparin-adjusted samples were centrifuged at 2000 × *g* for 5 min at 4 °C. Plasma was transferred to new tubes and protein concentration was quantified via DC assay (Bio-Rad, #5000112). The plasma samples were stored at − 80 °C until needed for the *Gaussia* luciferase assay.

For tail bleed, mice were restrained into a mouse restrainer. Animals were deeply anesthetized and placed into the stereotaxic device. Tail vein was nicked with a scalpel and approximately 80–100 µL of blood was collected in pre-weighed 1.5 mL centrifuge tubes that contained 20 µL of heparin (Sagent Pharmaceuticals). Blood samples were processed as described above.

### Organ sampling and protein extraction

Mice were perfused with PBS. Brain, heart, lung, liver, kidney, spleen, and skeletal muscle were excised, and flash frozen in isopentane on dry ice. Punches were collected from organs and homogenized in RIPA buffer (pH 7.4; 50 mM Tris HCl, 0.25% Sodium Deoxycholate, 150 mM NaCl, 1 mM EDTA) supplemented with 1% NP40 and protease inhibitors (MilliporeSigma) immediately before use. Homogenates were rotated for 30 min then centrifuged for 10 min at 10,000 × *g* at 4 °C. The supernatant, containing the soluble protein, was transferred to new tubes and its protein concentration was quantified via DC assay (Bio-Rad). Alternatively, brains were mounted for sectioning with a cryostat (Leica CM1950). Punches of cortex, striatum, hippocampus, and lower midbrain corresponding to SNpc, and VTA were sampled and processed as described above. The protein samples were stored at − 80 °C until needed for the GLuc assay.

### *Gaussia* luciferase assay

After quantifying protein levels of plasma and tissue lysates via DC assay (Bio-Rad), 60 µg of plasma and 20 µg of tissue lysates were transferred into white 96-well plates in technical triplicates. Each sample was brought up to 10 µL with PBS. The GLuc substrate was comprised of 8 µM coelenterazine (Regis Technologies) in PBS. Coelenterazine stock solutions were prepared at 20 mM in acidified methanol (10 μL of 10 M HCl in 1 mL of methanol) and stored at − 80 °C as single-use aliquots. The substrate was incubated at room temperature for 30 min prior to use. The luciferase assay was measured by a plate reader (Agilent, Synergy II) which measured the luciferase activity after ejecting 100 µL of substrate in each well. Luminescence was measured in relative light units.

### Statistical analysis

Statistical comparisons of data between the different groups were performed using a one-way and two-way ANOVA, followed by a Tukey’s post hoc multiple comparisons test. A value of *p* < 0.05 was considered significant.

## Results

**Fig. 1:**
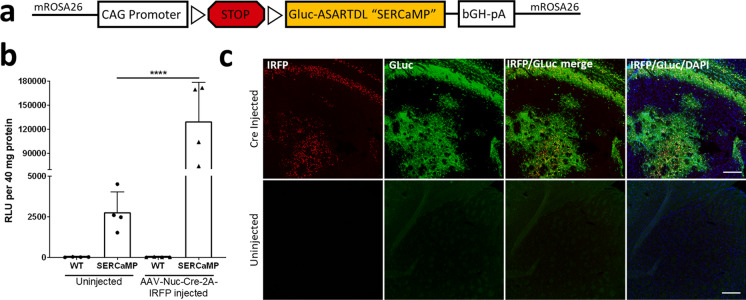
ER localized Cre-dependent GLuc expression construct. **a** Schematic of the GLuc-ASARTDL “SERCaMP” reporter. The reporter sequence was targeted to the mROSA26 locus and is preceded by a LoxP-STOP-LoxP (LSL) sequence. The reporter is under the transcriptional control of the CAG promoter. **b–c** Striatum of LSL-SERCaMP and wildtype (WT) mice were unilaterally injected with Cre recombinase packaged into AAV1-Nuc-IRFP-2A-iCre (2.25E + ^11^ vg/mL) to examine SERCaMP functionality. **b** GLuc activity was measured from homogenized AAV injected and uninjected striatal tissue samples. Two-way ANOVA with Tukey’s post-hoc comparison (^****^p < 0.0001). Data are presented as mean ± S.D. **c** GLuc immunohistochemistry was performed on mouse striatal brain sections. Scale bar 200 µm

We examined the specificity and efficacy of our GLuc-SERCaMP reporter by conducting a series of assays to confirm reporter expression and secretion into the extracellular space. A schematic of the construct used to target LSL-GLuc-ASARTDL or “SERCaMP” is shown (Fig. 1a) and additional information on the initial creation and characterization of SERCaMP has been reported previously (Henderson et al. 2014). We first tested animals for Cre-dependent expression of SERCaMP by injecting an AAV vector that co-expresses Cre recombinase and a nuclear localized near-infrared fluorescent protein (IRFP) into the striatum of LSL-SERCaMP mice and wildtype littermates. Two weeks after AAV-Nuc-IRFP-2A-iCre injection, striatal tissue from the uninjected and AAV-injected mouse brain hemispheres was assayed for GLuc activity. We observed significantly higher GLuc activity in the AAV-Nuc-2A-iCre injected hemisphere of LSL-SERCaMP mice (Fig. 1b) as compared to the uninjected hemisphere. The uninjected hemisphere of LSL-SERCaMP animals had low GLuc activity that could be attributed to the secreted GLuc present on the contralateral side. The IRFP signal overlapped with GLuc immunoreactivity in the injected hemisphere of SERCaMP brains (Fig. 1c).

**Fig. 2:**
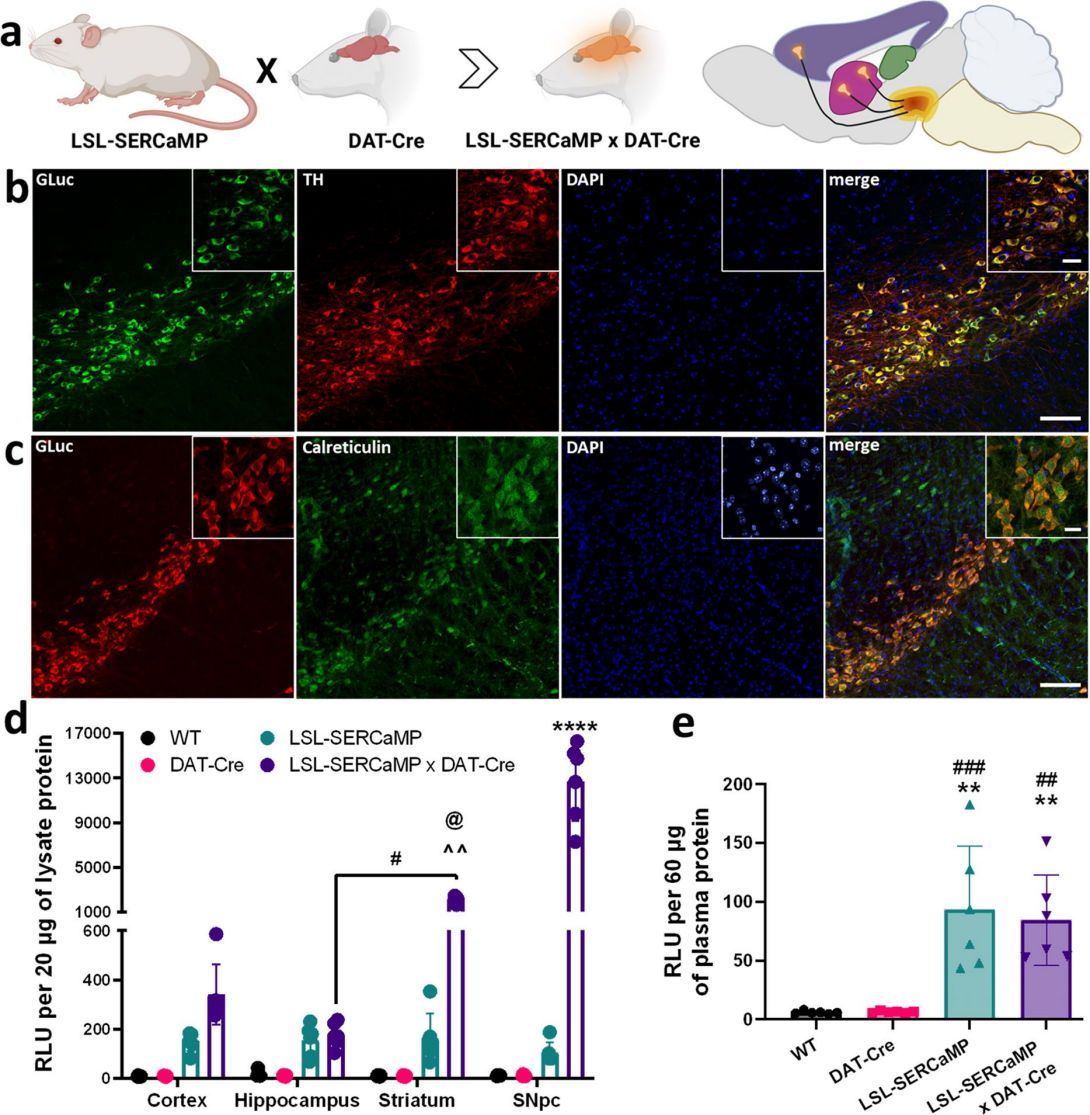
GLuc expression and activity is localized to the midbrain of LSL-SERCaMP × DAT-Cre mice. **a** Schematic representation of mouse crossing and sagittal representation of midbrain (yellow) dopaminergic neuron projections to cortex (purple) and striatum (pink). Created with BioRender.com. **b–c** SERCaMP reporter expression in brain was examined by immunohistochemistry. Brain sections containing substantia nigra region from LSL-SERCaMP × DAT-Cre mice were co-stained for GLuc and **b** tyrosine hydroxylase (TH) or **c** calreticulin. Scale bar 100 μm; inset 20 μm. **d** GLuc luminescence was measured from wildtype (WT), DAT-Cre, LSL-SERCaMP and LSL-SERCaMP × DAT-Cre mice, for cortical, striatal, hippocampal, and nigral brain tissue samples (n = 6). **** vs. all other columns, ^^ *vs.* WT and DAT-Cre mice, @ vs. LSL-SERCaMP. Two-way ANOVA with Tukey’s post-hoc comparison (^#,@^*p* < 0.05, ^^^^*p* < 0.01, ^****^p < 0.0001). Data are presented as mean ± S.D. **e** GLuc luminescence was measured from WT, DAT-Cre, LSL-SERCaMP and LSL-SERCaMP × DAT-Cre mice plasma. ^#^vs. WT, *vs. DAT-Cre (n = 6). One-way ANOVA with Tukey’s post-hoc comparison (^**,##^*p* < 0.01, ^###^*p* < 0.001). Data are presented as mean ± S.D

To examine cell-specific expression of our reporter we chose to drive expression in a well-defined and small cell population in the brain. The dopamine transporter is expressed in a subset of dopaminergic neurons in the mouse midbrain and the DAT-Cre mouse has been previously characterized (Bäckman et al. 2006). We confirmed that DAT-Cre restricts expression of Rosa26-targeted reporters by crossing DAT-Cre mice with the tdTomato Ai14 mouse line which contains a Cre-dependent LSL-tdTomato targeted to the Rosa26 locus. The tdTomato fluorescence was detected in midbrain neurons and striatal fibers, both of which colocalized with the dopaminergic cell marker tyrosine hydroxylase (TH) but not in cortical tissue (Supplementary Fig. S1). We crossed the LSL-SERCaMP mouse with the DAT-Cre mouse (Fig. 2a) and brain sections of the resulting LSL-SERCaMP × DAT-Cre mouse were double-immunolabeled for GLuc and either TH (Fig. 2b) or the luminal ER-resident protein, calreticulin (Fig. 2c). TH exhibited colocalization with GLuc in the *substantia nigra pars compact* (SNpc) and the ventral tegmental area (VTA; Fig. 2b) while GLuc colocalization with calreticulin demonstrated that the SERCaMP reporter was localized to the ER lumen (Fig. 2c). Wildtype mouse midbrain sections did not show similar colocalization with GLuc, when co-stained for TH (Supplementary Fig. S2a) or calreticulin, MANF, and TH (Supplementary Fig. S2b). Additionally, we examined GLuc immunostaining in the cortex, striatum, and hippocampus of LSL-SERCaMP × DAT-Cre mice (Supplementary Fig. S2c). GLuc was not detected in the cortex, striatum, or hippocampus despite the presence of dopaminergic cell projections from SNpc to the striatum and to a lesser extent the cortical areas. It is likely that the amount of GLuc in the striatum is not sufficient to be detectable by immunohistochemistry due to low signal-to-noise ratio. This is supported by a GLuc activity assay performed on tissue punches from different brain regions of LSL-SERCaMP × DAT-Cre mice showing that SNpc and striatal tissue lysates had significantly more GLuc activity than hippocampal tissue lysates (Fig. 2d). Dopaminergic neurons in the midbrain project to striatal and cortical brain areas, but not the hippocampus, thus, GLuc signal is expected in striatum but not hippocampus (Björklund and Dunnett 2007). Each brain region showed higher GLuc activity for the LSL-SERCaMP × DAT-Cre offspring compared to the LSL-SERCaMP, DAT-Cre, and wildtype controls (Fig. 2d). We also measured the GLuc activity in blood plasma (Fig. 2d–e) and demonstrated that levels between the LSL-SERCaMP × DAT-CRE and LSL-SERCaMP were not significantly different (Fig. 2e), indicating reporter expressed from midbrain dopamine neurons cannot efficiently cross the blood–brain-barrier.

**Fig. 3:**
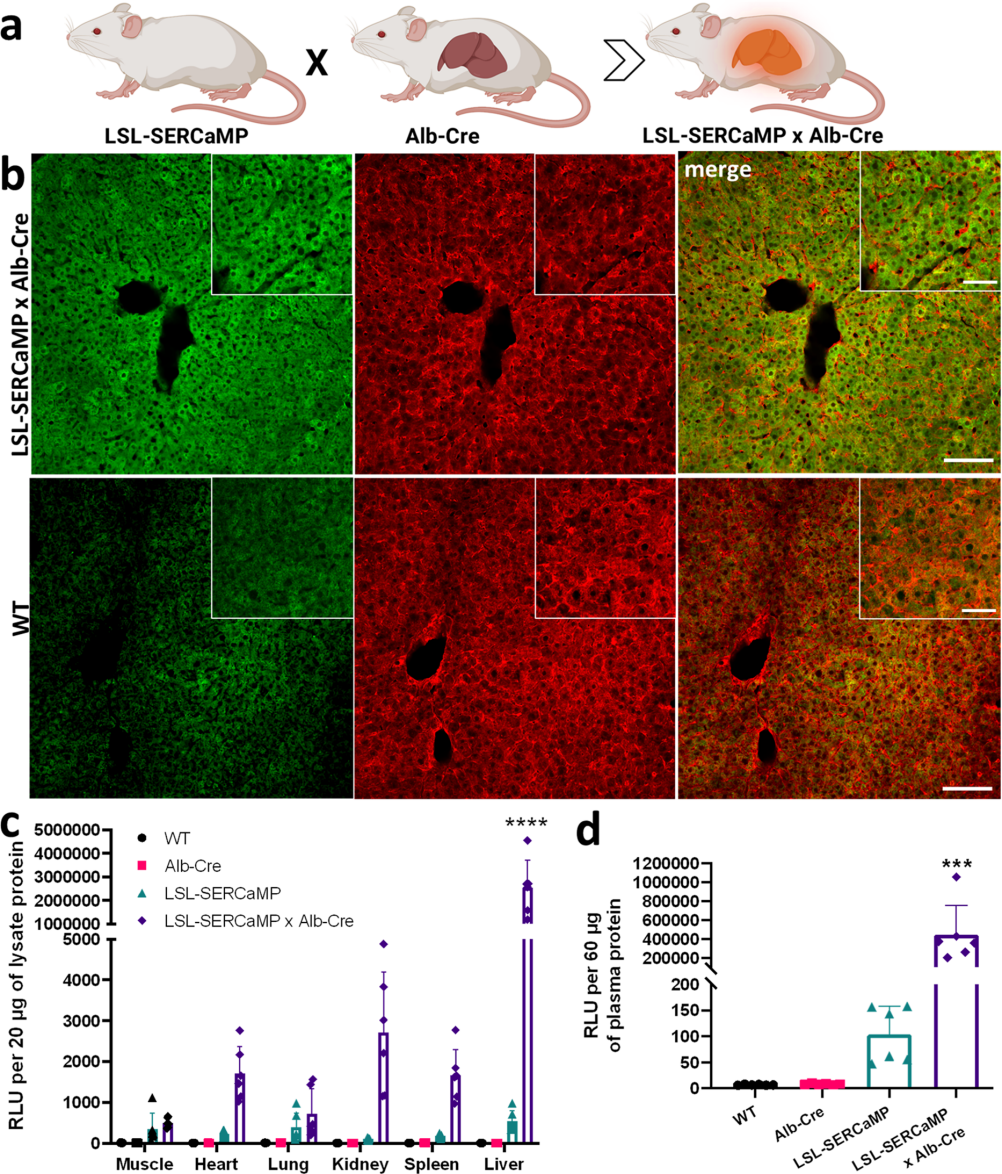
GLuc is detected in the plasma and liver of LSL-SERCaMP × Alb-Cre mice. **a** Schematic representation of mouse crossings. Created with BioRender.com. **b** LSL-SERCaMP × Alb-Cre or wildtype (WT) mouse liver samples were immunostained for GLuc and albumin. Scale bar 100 μm; inset 50 μm. **c** GLuc luminescence was measured from LSL-SERCaMP × Alb-Cre, LSL-SERCaMP, Alb-Cre and WT mice tissue homogenates (n = 5–6). Two-way ANOVA with Tukey’s post-hoc comparison (^****^p < 0.0001 *vs.* all other groups). Data are presented as mean ± S.D. **d** GLuc luminescence was measured from LSL-SERCaMP × Alb-Cre, LSL-SERCaMP, Alb-Cre and WT mice plasma. One-way ANOVA with Tukey’s post-hoc comparison (^***^p < 0.001 *vs.* all other groups)

The ability to monitor ER proteostasis longitudinally in the liver by simply sampling blood from the same animal would be an advantage of our new reporter mouse. We therefore crossed LSL-SERCaMP mice with an Alb-Cre mouse which should limit expression mostly to hepatocytes (Postic et al. 1999). As we did with the DAT-Cre cross, we first examined tdTomato expression from the LSL-tdTomato mouse crossed with Alb-Cre to test the expression of a reporter targeted to the Rosa26 locus. The tdTomato fluorescence was primarily localized to liver tissue (Supplementary Fig. S3). For comparison, there was no tdTomato fluorescence in the liver of DAT-Cre × LSL-tdTomato mice (Supplementary Fig. S2). We also examined brains of Alb-Cre × LSL-tdTomato and did not detect tdTomato fluorescence in midbrain (Supplementary Fig. S4). We observed a few vasculature-like and neuronal-like fluorescence patterns in other areas of the brain that have been reported (Supplementary Fig. S4); (Aldecoa et al. 2020). We next crossed the Alb-Cre with LSL-SERCaMP (Fig. 3a) and liver sections from offspring showed colocalization of GLuc and albumin immunolabeling while wildtype littermate sections did not have GLuc immunoreactivity (Fig. 3b). Tissue samples from liver, lung, heart, kidney, spleen, and muscle from LSL-SERCaMP × Alb-Cre, LSL-SERCaMP, Alb-Cre, and wildtype strains were assayed for GLuc activity (Fig. 3c). A significant difference in GLuc levels was detected in the LSL-SERCaMP × Alb-Cre mice livers when compared to muscle, heart, lung, kidney, or spleen of LSL-SERCaMP × Alb-Cre mouse as well as when compared to all tissue samples from LSL-SERCaMP, Alb-Cre, and wildtype mice. Blood plasma was collected from the same set of animals (Fig. 3d) and significantly more GLuc was detected in LSL-SERCaMP × Alb-Cre plasma when compared to LSL-SERCaMP, Alb-Cre, and wildtype mouse plasma samples.

**Fig. 4:**
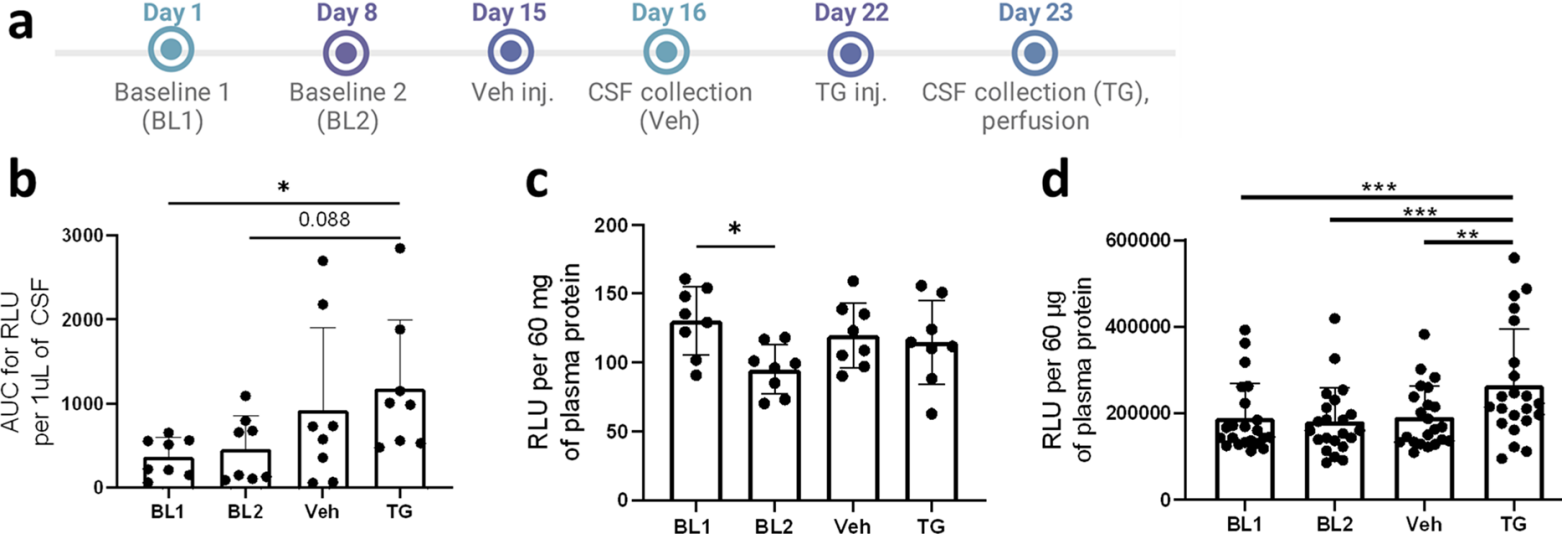
GLuc is increased in CSF and plasma after LSL-SERCaMP × DAT-Cre and LSL-SERCaMP × Alb-Cre, respectively, following treatment with thapsigargin (TG). **a** Experimental timeline for TG injection**.** Created with BioRender.com. **b–c** LSL-SERCaMP × DAT-Cre mouse CSF (**b**) and blood plasma **c** was collected at baseline (BL) and after vehicle and TG intracerebroventricular injection (n = 8). **d** GLuc luminescence was measured from LSL-SERCaMP × Alb-Cre mouse plasma at baseline and after i.p. injection of vehicle and TG (n = 22). Two-way ANOVA with Tukey’s post-hoc comparison, ^*^p < 0.05, ^**^p < 0.01, ^***^p < 0.001. Data are presented as mean ± S.D

The SERCaMP reporter is secreted in response to ER calcium depletion in vitro. To verify that the reporter responds to ER calcium depletion we used thapsigargin (TG), an irreversible SERCA pump inhibitor (timeline in Fig. 4a). We anticipated that depletion of ER calcium stores would lead to increased levels of GLuc activity in the extracellular fluids. GLuc signal was measured from LSL-SERCaMP × DAT-Cre mouse CSF (Fig. 4b) and blood plasma (Fig. 4c) or LSL-SERCaMP × Alb-Cre blood plasma (Fig. 4d). GLuc levels were increased in the CSF, but not the plasma of LSL-SERCaMP × DAT-Cre mice between baseline and post TG injection (Fig. 4b, c).

Individual mouse responses can be found in Supplementary Fig. S5. Due to the relatively low protein concentration in CSF, CSF samples were diluted in PBS containing 1 mg/mL BSA to prevent GLuc adhesion to the plate (Dijkema et al. 2021). We confirmed that CSF samples containing GLuc require a carrier protein (BSA) to prevent loss of signal during a plate-based assay (Supplementary Fig. S6). In LSL-SERCaMP × Alb-Cre mouse plasma, baseline GLuc levels normalized to plasma protein levels varied widely between individual mice, but GLuc levels were significantly increased after TG treatment (Fig. 4d). Individual mouse traces are shown in Supplementary Fig. S7.

## Discussion

Our lab has previously demonstrated that a subset of ER-resident proteins are secreted from the cell after ER calcium depletion (Trychta et al. 2018), but current tools to measure this phenomenon of exodosis in vivo are limited. Using our previously characterized reporter of exodosis, GLuc-SERCaMP (Henderson et al. 2014, Henderson et al. 2015), we created a Cre-dependent LSL-SERCaMP transgenic mouse line.

We demonstrate that GLuc-SERCaMP is expressed in a Cre-dependent manner in both brain and liver. Additionally, intracellular localization of GLuc-SERCaMP is restricted to the ER as shown by colocalization with calreticulin, an ER-resident protein. We observed some calreticulin nuclear localization, but it has been previously reported that in mice, calreticulin can facilitate nuclear export of nuclear receptors (Burns et al. 1994; Holaska et al. 2001).

Dopaminergic neurons represent a small cell population in mouse midbrain with approximately 20 000 cells and comprise the majority of DAT positive cells in the brain (Nelson et al. 1996). DAT expression outside the central nervous system has been reported in lymphocytes and detected in thymus and spleen (Amenta et al. 2001; Mignini et al. 2006). Our data show that plasma GLuc luminescence in LSL-SERCaMP × DAT-Cre mice is comparable to LSL-SERCaMP mice alone indicating that peripheral GLuc-SERCaMP secretion in SERCaMP × DAT-Cre mice is negligible under normal conditions. The low levels of GLuc activity in the blood also suggests that GLuc-SERCaMP secreted from midbrain dopaminergic neurons is not crossing the blood brain barrier (El-Amouri et al. 2013; Khasawneh et al. 2018). In the brain of LSL-SERCaMP × DAT-Cre animals, GLuc-SERCaMP activity was highest in the midbrain where the dopaminergic neuron cell bodies are located. The next highest activity was detected in the striatum where the projections and terminals from midbrain dopaminergic neurons are primarily located. GLuc activity was slightly elevated in the cortex which has few dopaminergic projections, but GLuc activity in hippocampus, where there are no known dopamine projections, did not rise above background levels found in LSL-SERCaMP mice. GLuc immunoreactivity in LSL-SERCaMP × DAT-Cre mice was readily detected in the dopaminergic neurons of the midbrain with minimal detection elsewhere. The lack of GLuc immunoreactivity in the striatal samples, despite GLuc enzymatic activity being detectable, is likely due to the higher signal to noise using a bioluminescence, compared to fluorescence, readout in the brain samples.

Hepatocytes represent a large cell population in the mouse periphery with an estimated 130 million cells per gram of mouse liver (Sohlenius-Sternbeck 2006; Rogers and Dintzis 2018). Analyses of LSL-SERCaMP × Alb-Cre liver tissue samples and plasma showed that SERCaMP is highly expressed in the liver and is secreted into the bloodstream with relative GLuc activity more than three to four orders of magnitude higher than in other genotypes or organs, supporting the liver is the primary source of SERCaMP in the LSL-SERCaMP × Alb-Cre.

We have previously observed that the rodent liver secretes SERCaMP reporter in response to thapsigargin. SERCaMP secretion is maintained for 72 h after TG administration and returns to baseline at 4 to 5 days (Henderson et al. 2014, Henderson, Wires et al. 2015). In the present study, ER calcium depletion caused by TG injection increased GLuc signal in the plasma and CSF of LSL-SERCaMP × Alb-Cre and LSL-SERCaMP × DAT-Cre mice, respectively. Even though we observed variation in the baseline signal between individual mice, GLuc signal after TG treatment was approximately 1.5 to 2 times higher when compared to baseline measurements of the same mouse. The range of the measured increase in GLuc signal after TG treatment is consistent with previous results observed in rats after hepatic AAV-SERCaMP injections (Wires et al. 2017). We collected blood 16 to 24 h post TG injection, because TG causes blood coagulation that makes it difficult to collect sufficient blood volume at 8 h post TG injection (Smeets et al. 1993). We did not see any increase in GLuc signal for the LSL-SERCaMP × DAT-Cre mice plasma after TG injection, but we did measure an increase in GLuc signal in CSF after TG administration. Our data support that the LSL-SERCaMP reporter mouse can be used to detect ER exodosis triggered by ER calcium depletion from a small and large populations of cells. The choice of the Cre driver line will affect the basal SERCaMP levels in the tissue and fluid being analyzed. Preliminary studies should be conducted to determine the basal expression in fluid of interest for each Cre driver crossed with LSL-SERCaMP.

The GLuc reporter is considered a very robust reporter, but it has a couple of limitations when using it with in vivo systems. For example, GLuc retains approximately 20% of its activity at 37 °C (Larionova et al. 2018) and even though GLuc flash kinetics produces an intense burst of light (Tannous et al. 2005), it was recently reported that GLuc is rapidly inactivated due to the formation of covalent product with its substrate coelenterazine (Dijkema et al. 2021). Thus, longitudinal in vivo imaging might be limited by the inactivation of circulating GLuc unless an alternative substrate can be identified. Additionally, the use of the CAG promoter, a relatively strong promoter, may provide a higher baseline secretion of the reporter that narrows the window of detecting stress-induced exodosis.

ER calcium depletion is observed in the development of many pathologies, including neurodegenerative (Parkinson’s, Alzheimer’s disease, Amyotrophic Lateral Sclerosis, and stroke), cardiovascular (atherosclerosis, heart failure, ischemia), and metabolic diseases (steatosis, alcoholic liver disease, type 2 diabetes) (Cabral-Miranda and Hetz 2018; Wang et al. 2018; Hetz et al. 2020). Additionally, diseases associated with SERCA pump and ryanodine receptor mutations show a more direct relationship between decreased ER calcium and pathology. For example, Brody myopathy characterized by mutations in SERCA1 leads to muscle weakness (Molenaar et al. 2020), Darier disease characterized by mutations in SERCA2 is associated with increased risk of heart failure, (Bachar-Wikstrom et al. 2020) and ryanodine receptor 1 related myopathies are associated with muscle weakness and atrophy (Greer et al. 2022). It has been shown that high-fat diet can trigger exodosis in vivo (Wires et al. 2017), but little is known about exodosis in other pathological conditions in vivo. The LSL-SERCaMP mice would be a useful tool for evaluating temporal changes in exodosis in animal models of diseases associated with ER calcium depletion. Additionally, the GLuc-SERCaMP reporter was used to identify drugs that stabilize ER calcium depletion and exodosis (Henderson, Trychta et al. 2021) and the LSL-SERCaMP mouse could be used to evaluate the activity of such compounds in vivo.

In summary, a new transgenic mouse line with a Cre dependent ER exodosis reporter protein, GLuc-SERCaMP, allows for simple and repeated measurements from the same animal using extracellular fluid collection.

## Supplementary Information

Below is the link to the electronic supplementary material.

Supplementary file1 (PDF 560 KB)
