# Supplementary material for: A transgenic mouse line for assaying tissue-specific changes in endoplasmic reticulum proteostasis
Source: Transgenic Res. 2023 May 3;32(3):209–21. doi: 10.1007/s11248-023-00349-7 (PMC10195735; doi:10.1007/s11248-023-00349-7)
Supplement: Supplementary file 1 — Supplementary file1 (PDF 560 KB) [file 11248_2023_349_MOESM1_ESM.pdf]

Supplementary figures

Journal name: Transgenic Research

Title: **A new transgenic mouse line for assaying tissue-specific changes in endoplasmic reticulum proteostasis**

Authors: Reinis Svarcbahs[1], Sarah Blossom[1], Helena S. Baffoe-Bonnie[1], Kathleen A. Trychta[1], Lacey K. Greer[1], James Pickel[3], Mark J. Henderson[1,2], Brandon K. Harvey[1,4]

[1]Molecular Mechanisms of Cellular Stress and Inflammation Section, Intramural Research Program, National Institute on Drug Abuse, National Institutes of Health, Baltimore, MD 21224, USA.

[2]National Center for Advancing Translational Sciences, National Institutes of Health, Rockville, MD 20850, USA.

[3]Transgenic Technology Core, Intramural Research Program, National Institute of Mental Health, Bethesda, MD 20892, USA.

[4]Corresponding author: bharvey@mail.nih.gov

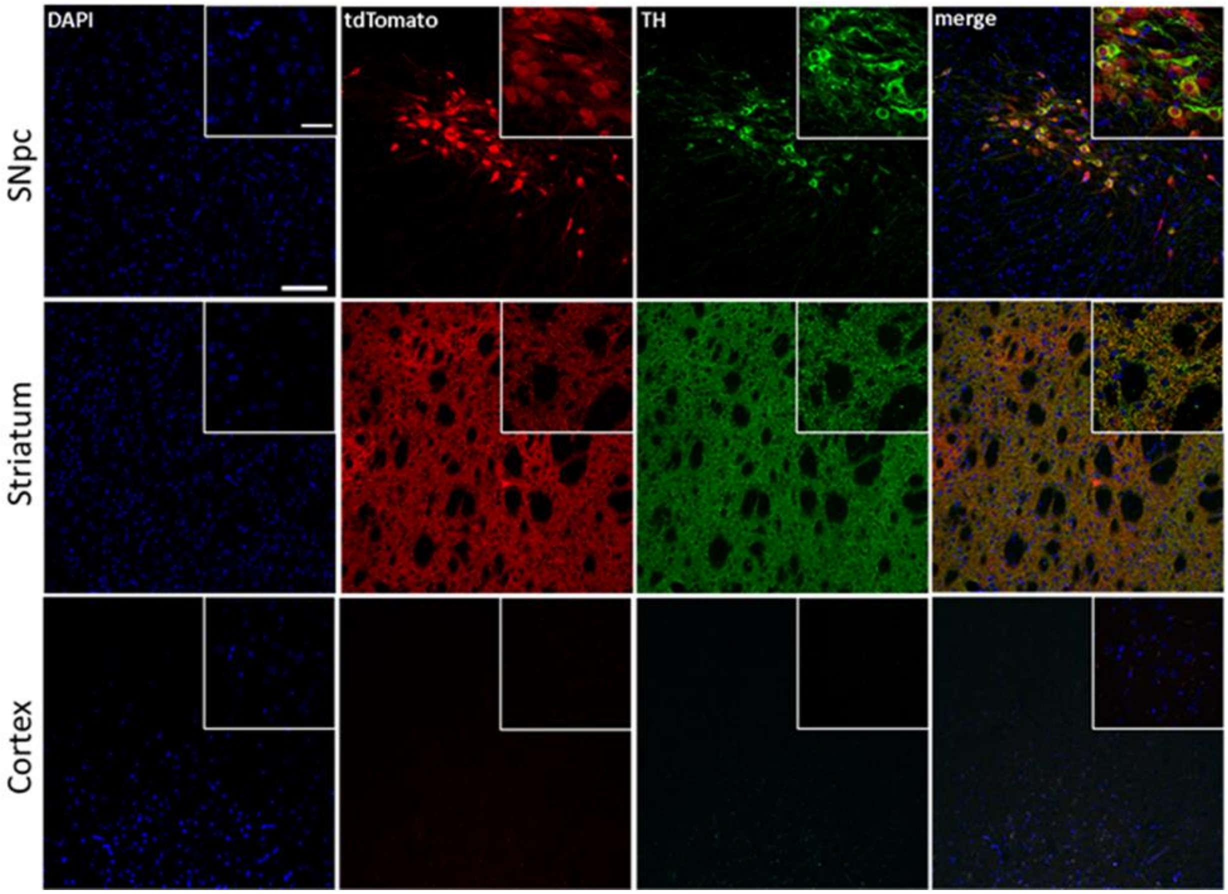

Supplementary Fig. 1 TdTomato x DAT-Cre colocalization in midbrain

TdTomato x DAT-Cre mouse brain tissue was immunostained for TH. Representative images from SNpc, Striatum and Cortex. Scale bar 100 μm; inset 50 μm.

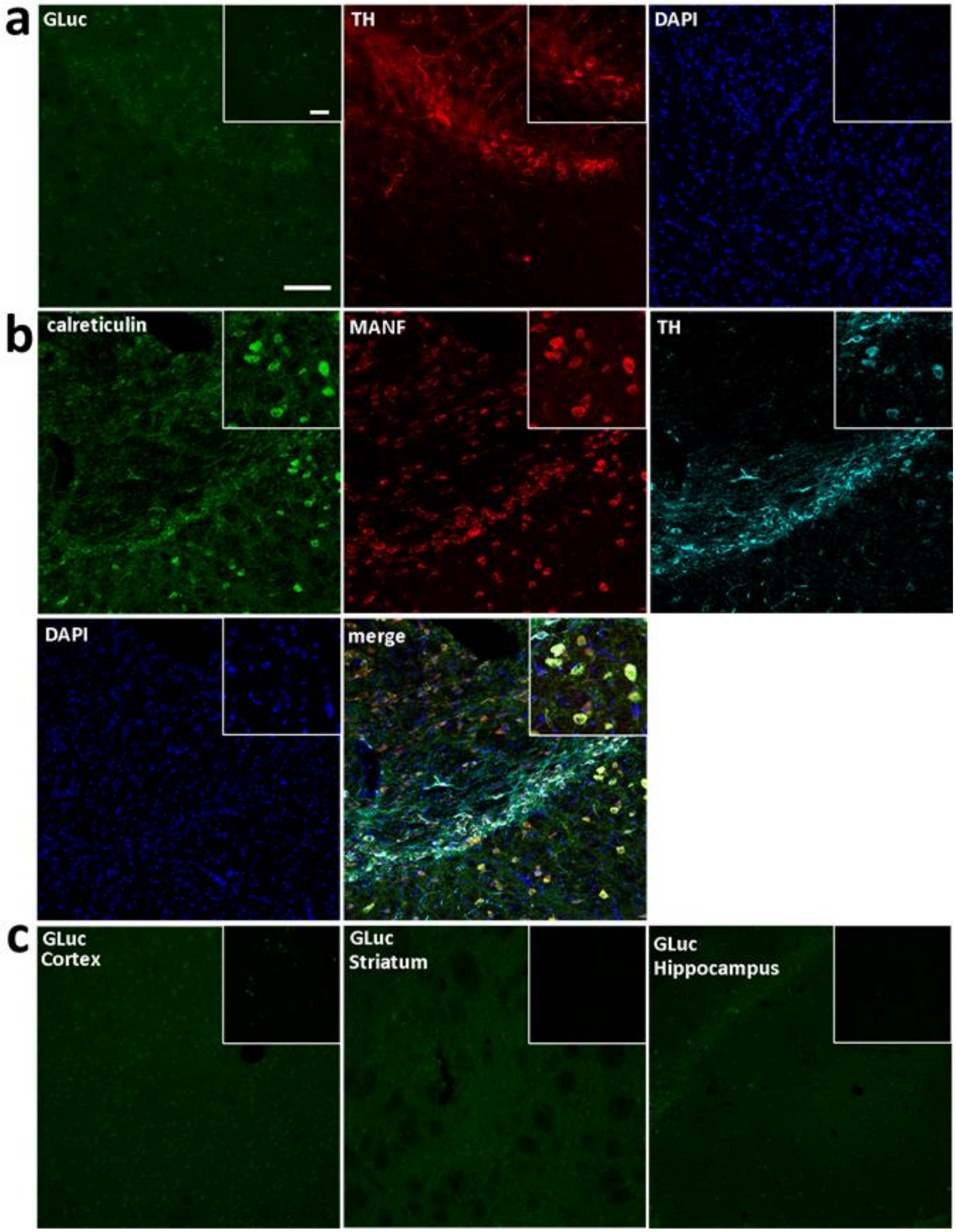

Supplementary Fig. 2 GLuc immunoreactivity

(**a**) Wildtype mouse SNpc brain sections were stained for GLuc and TH. (**b**) Wildtype mouse SNpc brain sections were stained for calreticulin, MANF, and TH. (**c**) The cortex, striatum, and

hippocampus of a LSL-SERCaMP x DAT-Cre mouse were immunostained for GLuc. Scale bar 100 µm; inset 50 µm.

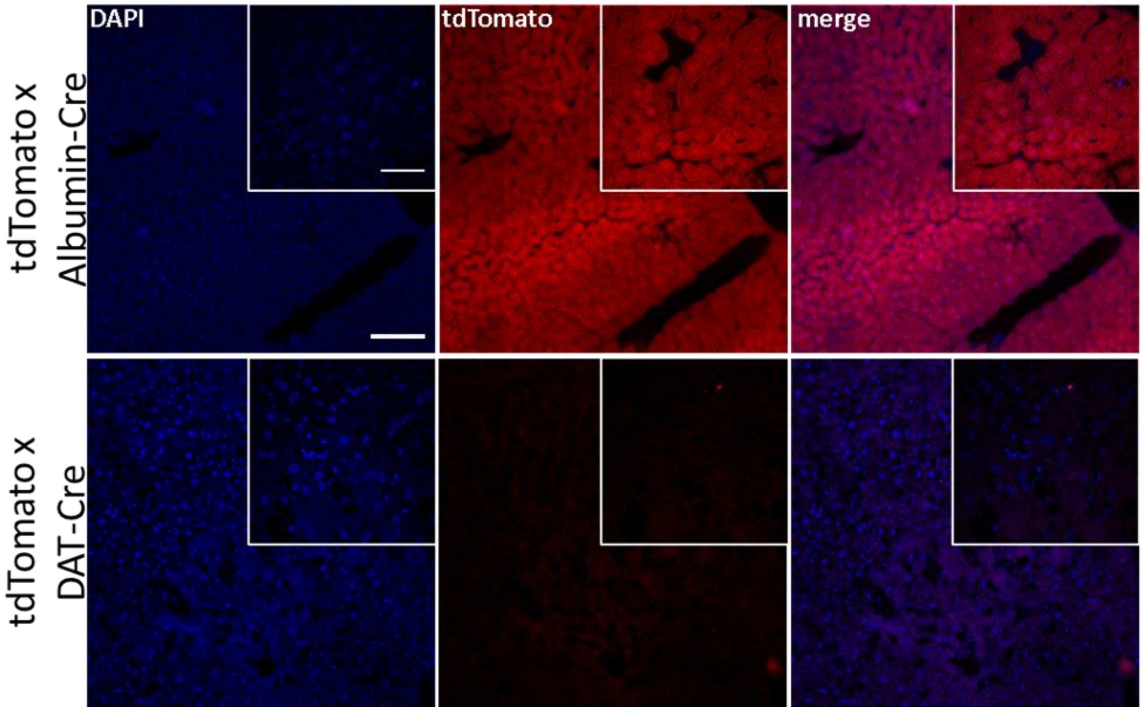

Supplementary Fig. 3 TdTomato x Alb-Cre localization in liver

TdTomato x Alb-Cre and tdTomato x DAT-Cre mouse liver tissue was imaged but only tdTomato x Alb-Cre showed fluorescence. Scale bar 100 µm; inset 50 µm.

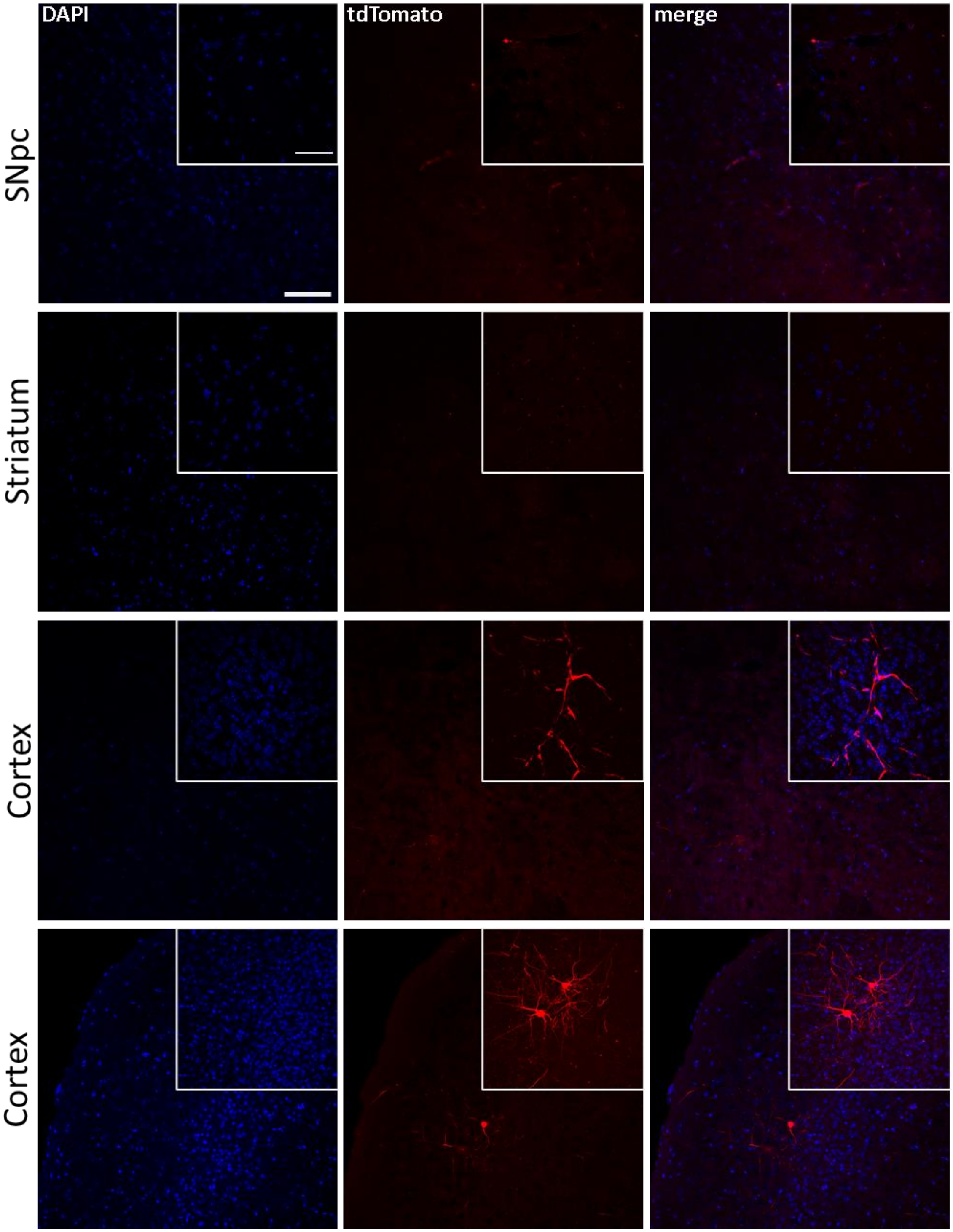

Supplementary Fig. 4 TdTomato x Alb-Cre localization in brain tissue

TdTomato x Alb-Cre mouse SNpc, Striatum and cortex was imaged. Vascular and glial structures were observed in brain tissue samples. Scale bar 100 µm; inset 50 µm.

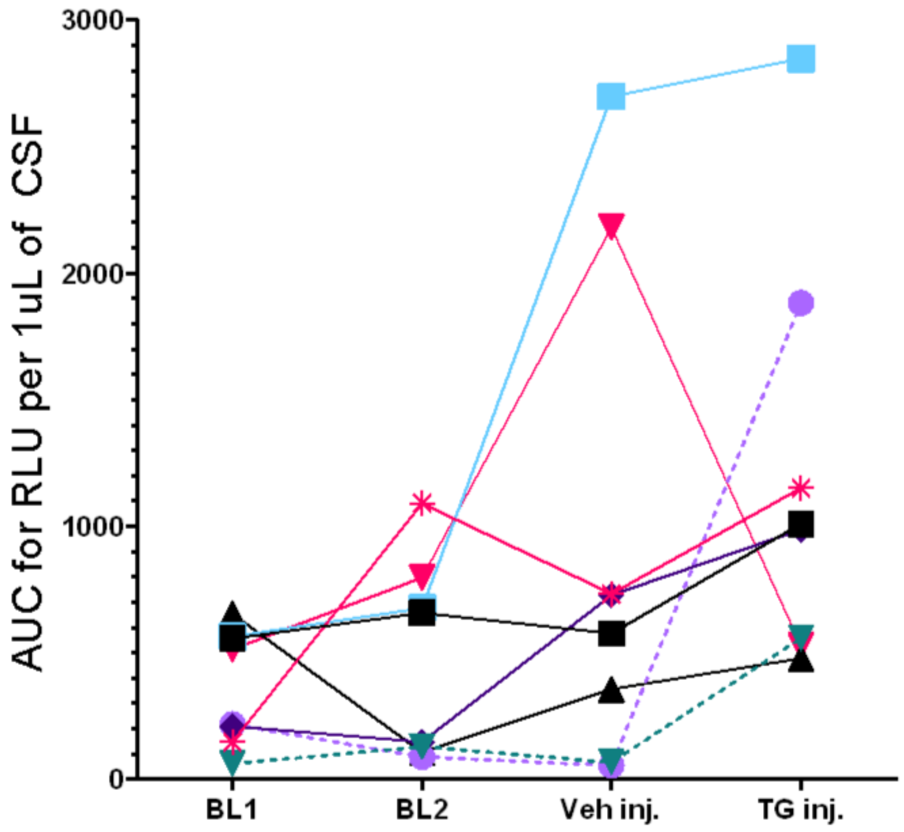

Supplementary Fig. 5 Line graph of individual mice from Fig. 4b

GLuc luminescence from LSL-SERCaMP x DAT-Cre mouse CSF. Dotted lines represent female mice.

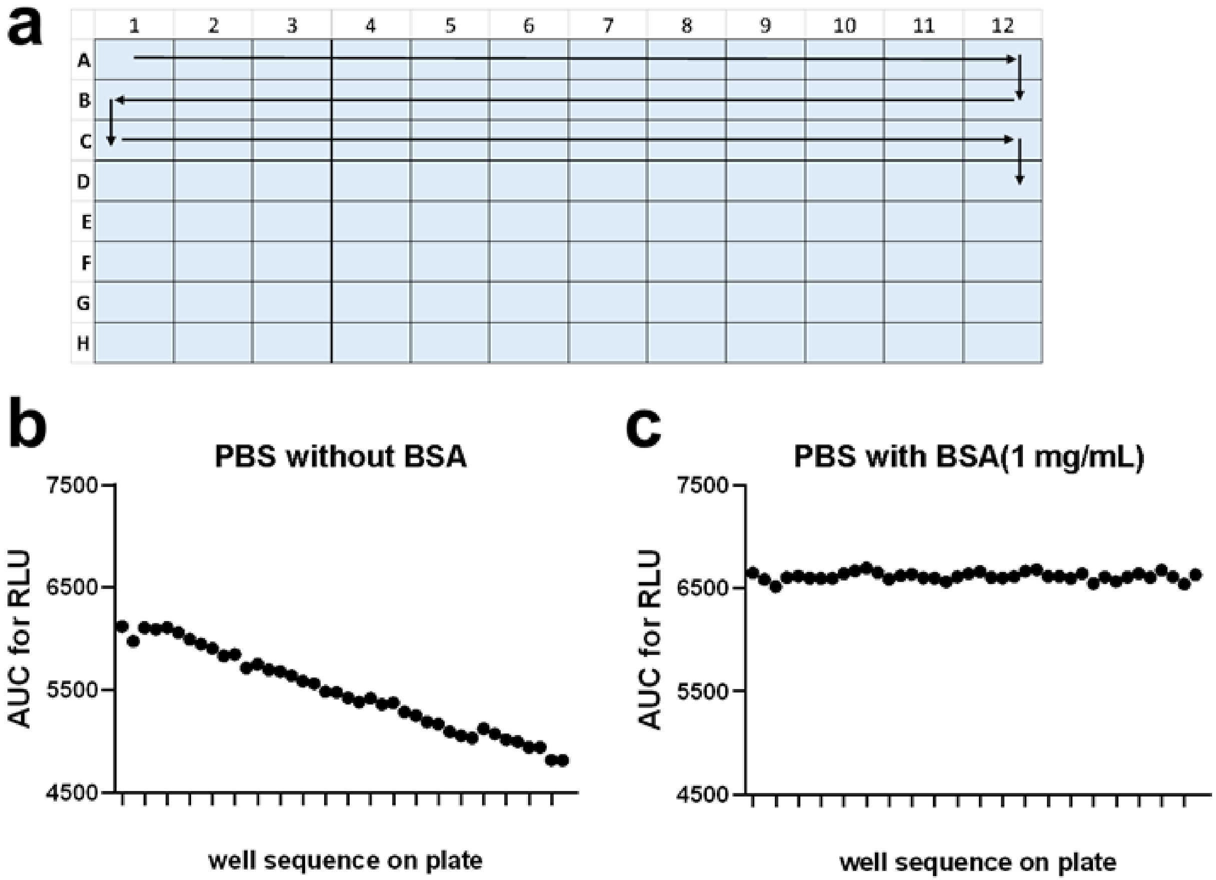

Supplementary Fig. 6 PBS and BSA role on low luminescence signal

(**a**) Serpentine pipetting path of the plate reader. (**b-c**) CSF sample with overexpressed SERCaMP was diluted in (**b**) PBS only or (**c**) PBS with 1 mg/mL BSA. 15 s kinetic measurement of the same sample was recorded on the plate. Data is presented as AUC for RLU for each individual well.

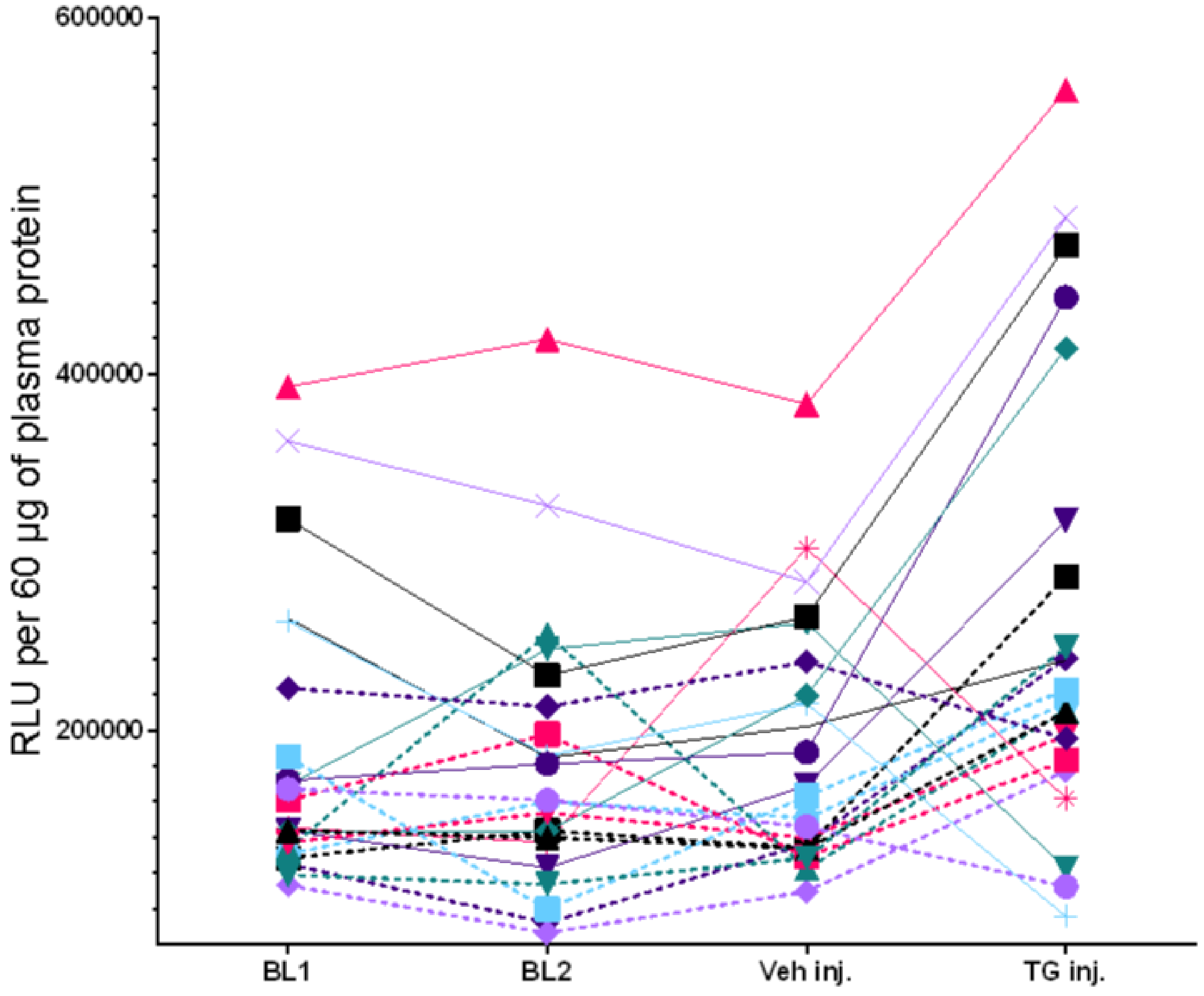

Supplementary Fig. 7 Line graph of individual mice from Fig. 4d

GLuc luminescence from LSL-SERCaMP x Alb-Cre mouse plasma. Dotted lines represent female mice.
